# Deferoxamine retinopathy: spectral domain-optical coherence tomography findings

**DOI:** 10.1186/1471-2415-14-88

**Published:** 2014-07-02

**Authors:** Cheng-Hsiu Wu, Chao-Ping Yang, Chi-Chun Lai, Wei-Chi Wu, Yi-Hsing Chen

**Affiliations:** 1 Department of Ophthalmology, Chang Gung Memorial Hospital, No. 5, Fu-Hsing Street, Kweishan, Taoyuan 333, Taiwan; 2 Department of Pediatrics, Chang Gung Memorial Hospital, Taoyuan, Taiwan; 3 College of Medicine, Chang Gung University, Taoyuan, Taiwan

**Keywords:** Spectral domain optical coherence tomography, Deferoxamine, Retinopathy

## Abstract

**Background:**

To describe the spectral domain optical coherence tomography (SD-OCT) findings of a patient who developed pigmentary retinopathy following high-dose deferoxamine administration.

**Case presentation:**

A 34-year-old man with thalassemia major complained of nyctalopia and decreased vision following high-dose intravenous deferoxamine to treat systemic iron overload. Fundus examination revealed multiple discrete hypo-pigmented lesions at the posterior pole and mid-peripheral retina. Recovery was partial following cessation of desferrioxamine six weeks later. A follow-up SD-OCT showed multiple accumulated hyper-reflective deposits primarily in the choroid, retina pigment epithelium (RPE), and inner segment and outer segment (IS/OS) junction.

**Conclusion:**

Deferoxamine retinopathy primarily targets the RPE–Bruch membrane–photoreceptor complex, extending from the peri-fovea to the peripheral retina with foveola sparing. An SD-OCT examination can serve as a simple, noninvasive tool for early detection and long-term follow-up.

## Background

Deferoxamine is an iron-chelating agent used to treat chronic iron overload in patients with thalassemia major and other hematologic conditions requiring routine blood transfusion [1,2]. The incidence of deferoxamine-related ocular toxicity is approximately 1.2% based on a prior study [3]. The clinical presentations may include night blindness, centrocaecal scotoma, constricted peripheral visual field, pigmentary retinopathy, and optic neuropathy [3]. Retinal pigmentary change was most frequently reported [4]. This case report pathologically characterizes the spectral domain optical coherence tomography (SD-OCT, SPECTRALIS SD-OCT, Heidelberg, Germany) and near-infrared reflectance (NIR) findings in a patient with deferoxamine retinopathy.

## Case presentation

**Figure 1:**
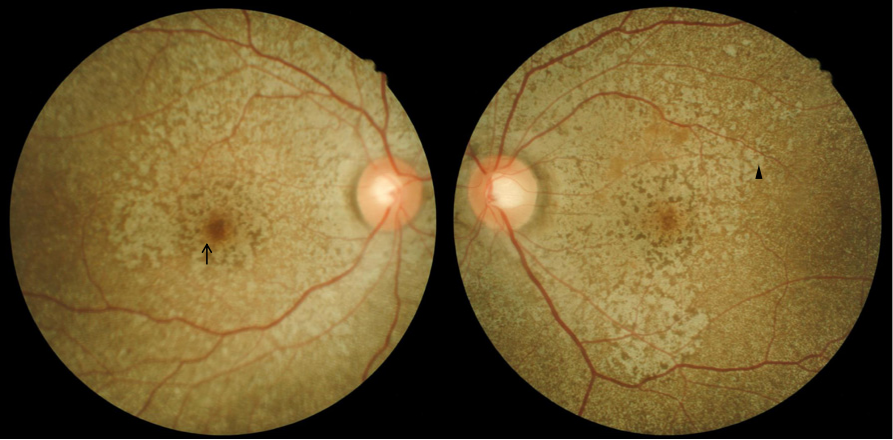
**Dilated fundus examination at the 6-week follow-up visit.** A dilated fundus examination revealed diffuse and confluent hypo-pigmented pinpoint lesions extending from the posterior pole (arrow) to the peripheral retina (arrowhead).

**Figure 2:**
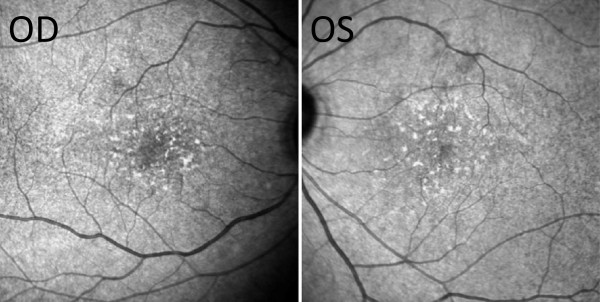
**Near-infrared reflectance (NIR) at the 6-week re-examination.** NIR showed hyper-reflective deposits particularly in the parafoveal and perifoveal areas.

**Figure 3:**
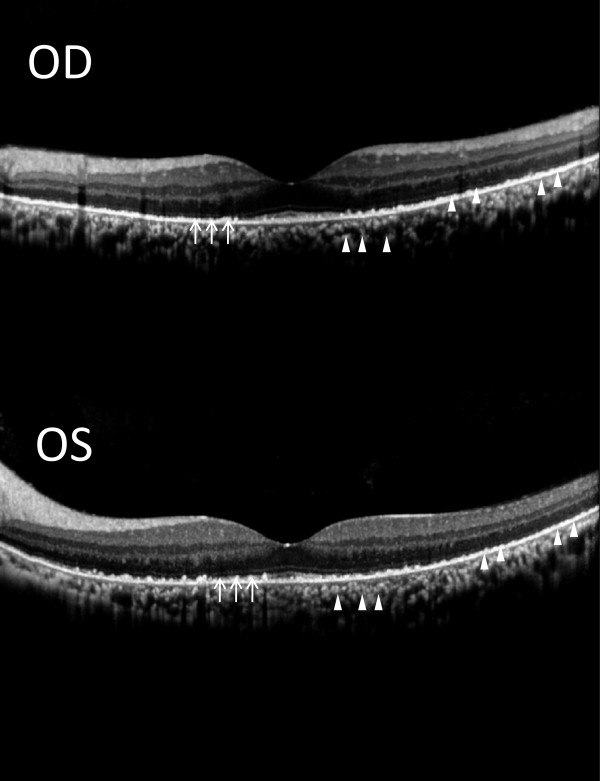
**SD-OCT at the 6-week re-examination.** SD-OCT revealed a disrupted IS/OS junction (arrow) and multiple diffuse and confluent hyper-reflective deposits in the retinal pigment epithelium (RPE), IS/OS junction, and choroid (arrowhead). Thickened RPE, Bruch’s membrane, and choroidal space were also observed.

A 34-year-old Taiwanese man with beta-thalassemia major had been administered routine blood transfusion and subcutaneous deferoxamine at 30 mg/kg/day for 20 years since youth. He was hospitalized for a compression fracture and myelopathy of the thoracic spine. He presented with acute onset of decreased vision, impaired color vision, and night blindness following continuous intravenous deferoxamine (98 mg/kg) for 42 days for the treatment of elevated serum ferritin level. On ophthalmic examinations, the best-corrected vision was 20/200 in the right eye and 20/40 in the left eye. The intraocular pressure measurement and anterior segment examination yielded normal results for both eyes. The fundus examination revealed multiple discrete hypo-pigmented circular lesions over the posterior pole and mid-peripheral retina in both eyes.Deferoxamine retinopathy was suspected, and the patient was switched to oral deferasirox/deferiprone. Six weeks later, there was an improvement in the best-corrected vision (20/60 in the right eye and 20/25 in the left eye) and color vision. Retinal pigmentary changes became confluent (Figure 1). NIR showed hyper-reflective deposits particularly in the parafoveal and perifoveal areas (Figure 2). SD-OCT showed multiple confluent hyper-reflective deposits in the choroid, retinal pigment epithelium (RPE) and IS/OS junction. Thickened RPE, Bruch’s membrane, and choroid space were also discovered. The IS/OS junction was most severely disrupted at the perifoveal and parafoveal areas than at the foveola area (Figure 3).

## Conclusion

Deferoxamine is a widely used chelating agent in treating transfusional hemochromatosis [1,5]. Visual symptoms included decreased visual acuity, night blindness, and colour vision abnormalities [2-6]. These ophthalmic examination findings have been reported extensively. Sumu et al. observed speckled hyper-fluorescence with well-demarcated areas of blocked fluorescence on fluorescein angiography [6]. Markedly reduced photopic, scotopic, and 30-Hz flicker response amplitudes were reported on electroretinograms. Electro-oculogram typically showed reduced light-peak to dark-trough ratios [4,6]. Viola et al. reported abnormal fundus autofluorescence in 9% of 197 patients, but only 5 patients reported visual symptoms [7]. Viola et al. further described the pattern dystrophy–like or minimal changes of macular lesions in patients with deferoxamine retinopathy by using NIR and SD-OCT which pointed out the disease itself affects the RPE–Bruch membrane–photoreceptor complex [8].

The pathophysiology of deferoxamine-related retinopathy has been investigated for several years. Rahi et al. first reported electron microscopic findings of deferoxamine retinopathy, including patchy RPE depigmentation, abnormally thickened Bruch's membrane, and normal photoreceptors [9]. Previous studies also discovered that iron overload and iron-chelating agents both may be mutually confounding factors in the causation of ocular changes of thalassemia such as RPE mottling [5,10-13]. The SD-OCT findings in our case revealed multiple confluent hyper-reflective deposits in the RPE, IS/OS junction and choroid (Figure 3). We hypothesized that hyper-reflective deposits detected by means of SD-OCT may represent a primarily involvement of RPE–Bruch membrane–photoreceptor complex in deferoxamine toxicity which correlated with previous histologic findings [8,9].

Ocular deferoxamine toxicity could cause vision impairment; regular ophthalmic assessment is required in these patients. We presented the SD-OCT findings of deferoxamine retinopathy highly correlated with previous histologic descriptions and showed that the toxicity primarily involved the RPE–Bruch membrane–photoreceptor complex. Noninvasive SD-OCT and NIR imaging, both well tolerated by patients, may be helpful in early detection and long-term monitoring.

### Consent

The patient provided written informed consent for the publication of this case report and any accompanying images. A copy of the written consent is available for editorial review.

## Abbreviations

SD-OCT: Spectral domain optical coherence tomography; IS/OS: Inner segment/outer segment; RPE: Retinal pigment epithelium.

## Competing interests

The authors declare that they have no competing interests.

## Authors’ contributions

C-HW conducted the literature search and composed the manuscript. Y-HC conceived the idea for the manuscript, conducted a literature search, and critiqued the manuscript. All authors read and approved the final manuscript.

## Pre-publication history

The pre-publication history for this paper can be accessed here:

http://www.biomedcentral.com/1471-2415/14/88/prepub
